# Evidence to support the mechanical advantage hypothesis of grasping at low force levels

**DOI:** 10.1038/s41598-022-25351-7

**Published:** 2022-12-02

**Authors:** Banuvathy Rajakumar, S. K. M. Varadhan

**Affiliations:** Department of Applied Mechanics, Indian Institute of Technology Madras, Chennai, 600036 India

**Keywords:** Bone quality and biomechanics, Motor control, Physiology

## Abstract

Grasping an object is one of the several tasks performed by human hands. Object stabilization while grasping is a fundamental aspect to consider for the safety of grasped objects. Fingertip forces re-distribute to establish equilibrium when systematic variations are introduced to objects held in hand. During torque variations to the grasped handle, the central nervous system prefers to support the mechanical advantage hypothesis. According to this hypothesis, during torque production tasks, fingers with longer moment arm for normal force produce greater normal force than the fingers with shorter moment arm. The current study was performed to examine and confirm the factor that causes the central nervous system to employ this strategy. In addition to minimising the thumb’s contribution to hold the handle, thumb normal force was restricted to a minimal level. Such a restriction made the task even more challenging. Therefore, it was confirmed that the challenging task induces the central nervous system to employ the mechanical advantage principle.

## Introduction

Human hands play a vital role in accomplishing a multitude of daily life activities, from object manipulation to exploration. Grasping is one common activity performed with the human hands of all healthy individuals. Object stabilization while grasping is the foremost important aspect to be considered for safe manipulation. Maintaining the hand-held object in static equilibrium by holding the object steady in air using hand is object stabilization. Fingertip forces of the individual fingers finely adjust to maintain the handle in static equilibrium.

The force distribution of the individual fingers was studied when the mass^1^, torque^2^, fingertip position^3^, and surface friction^4,5^ of the object were varied systematically. During any torque changes to the handheld object, our central nervous system prefers to make use of the mechanical advantage of the fingers to minimize the total effort (or force)^6^. According to the mechanical advantage hypothesis (MAH), during the moment production grasping tasks, considering thumb as a pivot point, peripheral fingers (index and little) with longer moment arms for the normal force produce greater normal force than the central fingers (middle and ring) with shorter moment arms for normal force. In the past, there were studies performed with five fingers prehensile handles to investigate the applicability of the mechanical advantage hypothesis.

In a study on the prehensile handle, load and torque changes were introduced to the handle by suspending loads of different masses at various distances from the handle's center of mass (COM)^7^. The instruction was to maintain the handle in static equilibrium. Due to the external torque changes, either index or little finger produced greater normal force than middle or ring finger depending on the torque direction. Thus, supporting the mechanical advantage hypothesis. Further, MAH was also supported in the study that involved an accurate handle rotation task involving five digits of the human hand^8,9^. In a multi-finger torque production study^6^, the use of mechanical advantage was investigated on a mechanically fixed and free handle. The results of the study supported the idea that the central nervous system utilizes the mechanical advantage during torque production in both fixed and free objects.

Further, our preliminary study on the five-fingers prehensile handle examined the mechanical advantage hypothesis when torque changes were introduced by placing the thumb on a slider (or unsteady) platform mounted over a vertical railing fitted on the handle frame^10^. Due to the unsteady thumb platform, the tangential force contribution of the thumb was constant and low, thus resulting in the pronation torque. As the instruction was to maintain the handle in static equilibrium, a compensatory supination torque was required. Thus, in the absence of mechanical constraint to fix the platform, the normal force of the ulnar fingers increased to produce the compensatory supination torque. The expectation was that, during the compensatory torque production, the little finger would exert greater normal force than the ring finger. In contrast to our expectation, ulnar fingers exerted statistically comparable normal forces when the unsteady thumb platform was held steady at the HOME position (midway between middle and ring fingers).

The mechanical advantage hypothesis was partially supported by a study involving moment production on a mechanically fixed vertically oriented handle^11^. It was assumed that the applicability of MAH is limited and specific to task and effector. Since there was no supportive evidence to explain this, we attempted to examine whether the applicability of the mechanical advantage principle is specific to any particular task and investigate the kind of task that lends support to MAH. To investigate this, our previous study involved systematically increasing the mass of the handle by adding external loads of mass 0.150 kg, 0.250 kg, 0.350 kg, and 0.450 kg^12^. With the addition of external loads, the magnitude of supination torque requirement also increased. The expectation was that MAH would be supported with the addition of external load. However, MAH was supported only when an external load of a greater mass of 0.450 kg was added. Since the thumb contribution to hold the handle was restricted to constant low magnitude, the other fingers were required to share the increasing load. Therefore, we believed that the difficulty associated with the task of maintaining the handle in static equilibrium with a larger external load of mass 0.450 kg, could be the reason for MAH to be supported.

There are different ways by which a task can be made challenging or difficult. It can be done by increasing the mass, reducing the surface friction of the grasped object, suspending a larger external load at a greater distance from the center of mass of the handle, and operating the fingers or thumb beyond their restricted range of motion. These situations demand greater normal force to be produced by the fingers and thumb for the successful completion of the task. Also, it is possible to make the task more difficult by imposing restrictions on the normal force produced by the thumb.

In the previous study, with an addition of greater external load (0.450 kg), which in turn resulted in the exertion of very high normal force by the thumb (around 16.50 N), had made the task more demanding. It is not only by producing very high normal force by thumb but also by restricting the thumb to produce lesser normal force (i.e. closer to the mass of the handle) could make the task demanding. Therefore, in the current study, in addition to the constraints of constant and low tangential force, minimal or no movement of a slider platform, an additional constraint of producing minimal normal force by the thumb was imposed. By this way, the task of maintaining the static equilibrium of the handle was made quite difficult to perform. In such a situation, the expectation was that the central nervous system (CNS) might prefer to use the little finger's mechanical advantage by producing greater normal force than the ring finger to complete the task successfully.

Thus, we hypothesized that CNS utilizes the mechanical advantage when the task is made demanding by instructing them to produce minimal thumb normal force (low force level grasp) while holding the handle with an unsteady platform (Hypothesis 1).

## Methods and materials

### Participants

Twelve right-handed male participants participated in this experiment. The mean and the standard deviation of height, weight, hand length, and width of the participants were age: 26.66 ± 3.22 years, height: 171.33 ± 7.54 cm, weight: 76 ± 13.17 kg, hand-length: 19.31 ± 0.70 cm, and hand-width: 9.02 ± 0.42 cm. Participants with no history of musculoskeletal injuries and neurological diseases were chosen to participate.

### Ethics approval

The experimental procedures were approved by the Institutional Ethics committee of the Indian Institute of Technology Madras (Approval Number: IEC/2021-01/SKM/02/05). All the participants gave written informed consent according to the procedure approved by the institutional ethics committee of IIT Madras before starting the experiment.. All experiments were conducted in accordance with relevant guidelines and regulations approved by the Institutional Ethics Committee of the Indian Institute of Technology Madras.

### Experimental setup

**Figure 1:**
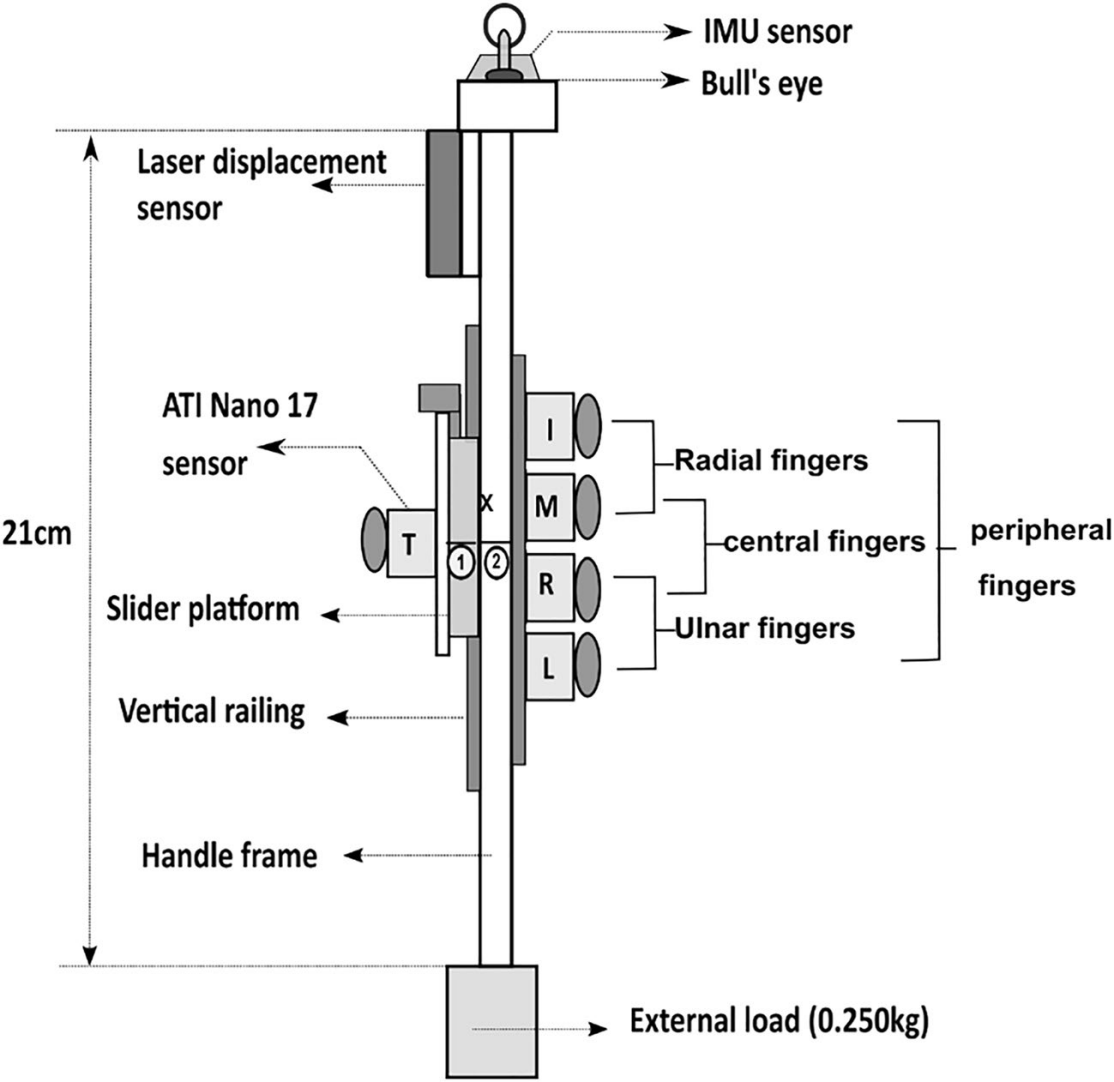
Schematic diagram of the experimental handle. The figure shows the schematic diagram of the experimental handle with the slider platform on the thumb side of the handle. The handle was made of an aluminum handle frame (21 × 1 × 3) cm with a slider platform mounted over the vertical railing of length 13.6 cm. The mass of the slider platform was 0.100 kg. Two horizontal lines were drawn on the participant side of the handle: one on the platform (referred by no:1) and another on the handle frame (referred by no:2). Five six axis force/torque sensors (ATI Nano 17) were mounted on the handle frame to measure the fingertip forces of the individual fingers and thumb. A displacement sensor and an IMU sensor were placed on top of the handle. An external load of mass 0.250 kg was attached at the bottom of the handle. The mass of the handle including slider platform and external load was 0.700 kg**.** The distance between the sensor surface of the thumb and other fingers (grip aperture) is 6.2 cm.

A five-finger instrumented prehensile handle was designed and built with a vertical railing of length 13.6 cm on the thumb side of the handle frame (see Fig. 1). A slider platform was mounted on the railing to translate in the vertical direction over the railing. The mass of the slider platform was 0.100 kg. The handle with slider platform was suspended from the top of wooden support using a nylon rope housed within a PVC pipe to prevent unnecessary lateral movements. The total mass of the handle, including the slider platform, was 0.450 kg. Five six-axis force/torque sensors (Nano 17, Force resolution: Tangential: 0.0125 N, Normal: 0.0125 N, ATI Industrial Automation, NC, USA) were mounted on the handle to measure the forces and moments exerted by the individual fingers and thumb. The force sensor for the thumb alone was placed on the slider platform. Soft finger contact model has been assumed and there is no influence of finger surface curvature on the direction of normal force generated.

An acrylic block was placed in the anterior–posterior direction on top of the handle. An intelligent 9-axis absolute orientation sensor (Resolution: 16bits, Range: 2000°/s, Model: BNO055, BOSCH, Germany) was placed on the acrylic block towards the monitor side. This IMU (Inertial Measurement Unit) measured the position and orientation of the handle during the experiment. Further, on top of the handle, towards the thumb side, a square acrylic piece was fitted to mount a laser displacement sensor (resolution, 5 μm; OADM 12U6460, Baumer, India). This displacement sensor was mounted to measure the displacement data of the thumb platform in the vertical direction while it translated along the vertical railing. A spirit level with a bull’s eye was placed on the acrylic block towards the participant’s side to check whether the handle was vertically oriented.

Two horizontal lines were drawn on the participant’s side of the handle, one at the center of the thumb platform (referred by no.1 in Fig. 1) and another line drawn midway between the middle and ring fingers (represents ‘HOME’ position) on the handle frame (referred by no.2 in Fig. 1). The participants were instructed to place the unsteady thumb platform by precisely aligning both lines while holding the handle. Thirty analog signals from the force/torque sensors (5 sensors × 6 components) and single-channel analog laser displacement data were digitized using NI USB 6225 and 6002 at 16-bit resolution (National Instruments, Austin, TX, USA). This data was synchronized with four channels of processed, digital data from the IMU sensor.

### Experimental procedure

Participants were asked to wash their hands with soap and towel dry before the start of the experiment. The right upper arm was abducted approximately 45° in the frontal plane, flexed 45° in the sagittal plane with the elbow flexed at approximately 90°. The natural grasping position can be achieved by supinating the forearm at 90°. The movements of the forearm and wrist were constrained by fastening with a velcro strap to the tabletop. The type of grasp that has been employed for holding the five finger prehension handle was the prismatic precision grip.

**Figure 2:**
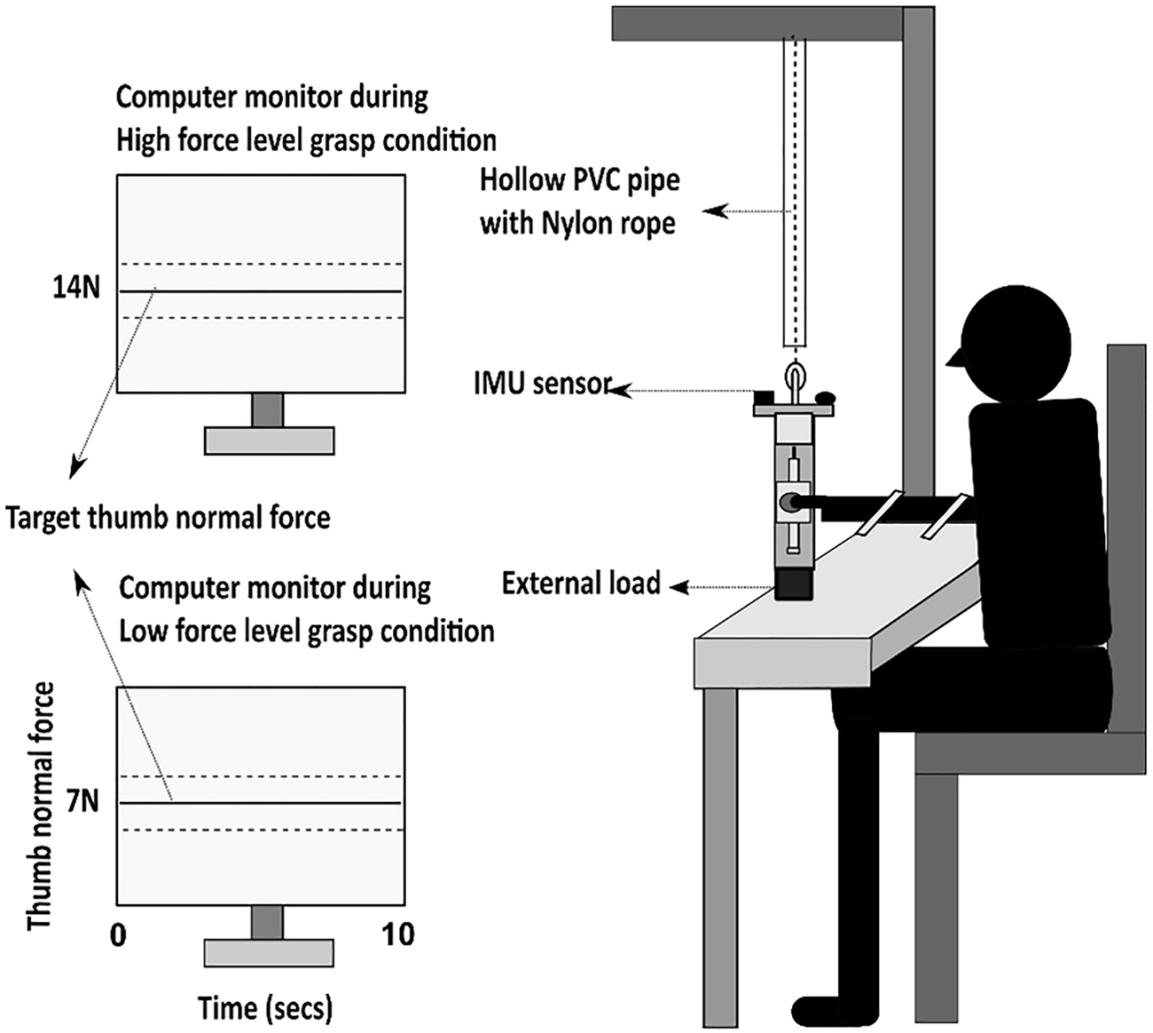
Schematic diagram of the experimental setup. The figure shows the experimental setup with a participant holding the experimental handle in front of the computer monitor. The monitor displayed a solid horizontal line that corresponded to the target normal force to be produced by the thumb. During high force level grasp condition, the solid horizontal line shown on the monitor corresponded to 14 N of normal force to be produced by the thumb. Whereas during low force level grasp condition, the solid horizontal line shown on the monitor corresponded to 7 N of normal force to be produced by the thumb. The two dashed lines above and below the solid line signify an error margin of ± 0.5 N.

The experiment consisted of two conditions: high force level grasp and low force level grasp. During both the conditions, the task was to maintain the handle in static equilibrium by holding the slider platform steady at the HOME position. Apart from this, in high force level grasp condition, the target thumb's normal force was set to 14 N. The participant’s computer monitor displayed only the solid horizontal target line corresponding to the target thumb normal force with two dashed lines, one above and below the solid line representing an error margin of ± 0.5 N (see Fig. 2). The participants were instructed to hold the platform steady by producing a thumb normal force which was shown as a visual feedback line to trace the solid horizontal target line. The trial was accepted only when the thumb’s normal force’s feedback line was within the acceptable error margin. Thus, the task of producing thumb normal force of 14 N matching the target normal force line had to be performed by precisely aligning the horizontal line on the thumb platform to the line drawn on the handle frame.

In low force level grasp condition, the target thumb normal force was set to 7 N. The participants were instructed to produce a minimal thumb normal force of 7 N, which would be fed as a feedback line to trace the target line corresponding to 7 N. This tracing task had to be performed by aligning the horizontal line on the thumb platform to the line drawn on the handle frame. The acceptable error margin of thumb displacement data for both conditions was ± 0.2 cm. Throughout the trial, in both conditions, the participants were instructed to avoid tilting the handle in any direction by maintaining the bubble at the center of the spirit level. The experimenter could view the thumb displacement data, net tilt angle (net rotation of the handle about all three axes (x, y and z) with reference to the source, measured in degrees), normal and tangential forces of the individual fingers and thumb on a separate computer monitor (not viewable by the participant).

For each experimental condition, twenty-five trials were provided. Each trial lasted 10 s. One minute break was provided between the trials. One hour break was provided between the conditions. Six participants performed high force level grasping in their first session, and the remaining six participants performed low force level grasping in their first session. In this way, the order of conditions was counterbalanced across participants.

### Data analysis

In each trial, the data between 3 and 7 s were taken for analysis to avoid start and end effects. The collected data were analyzed offline using MATLAB (Version R2016b, MathWorks, USA). Force/Torque data and laser displacement data of thumb were lowpass filtered at 15 Hz using second-order, zero phase lag Butterworth filter. The normal and tangential force data collected from the individual fingertips and the thumb were averaged over the time samples, trials, and participants for each condition separately, and the standard errors of the mean were computed.

### Statistics

All statistical analyses were performed using R. We performed a two-way repeated-measures ANOVA on the average normal force with the factors as *conditions* (2 levels: high force level grasp and low force level grasp) and *fingers* (4 levels: index, middle, ring, little). Since the thumb's normal force was dependent on the normal forces produced by index, middle, ring, and little fingers, the thumb was excluded from the ANOVA analysis. Sphericity test was done on the data, and the number of degrees of freedom was adjusted by Huynh–Feldt (H–F) criterion wherever required. We also performed pairwise post hoc Tukey tests to examine the significance within factors. An equivalence test was performed on the normal forces of the ulnar fingers (ring and little) collected during high force level grasp condition. The statistical equivalence was tested using the two one-sided t-tests (TOST) approach^13^ for a desired statistical power of 95%. The smallest effect size of interest (SESOI) was chosen as the equivalence bounds.

## Results

### Task performance

All the participants could trace the target normal force line during both conditions by producing appropriate thumb normal force within the error margin of ± 0.5 N. The root mean square error of the thumb normal force data was computed for high force level and low force level grasp conditions, shown in Table 1. Also, all the participants were able to produce the target force by aligning the horizontal line on the platform to the line drawn between middle and ring fingers within an acceptable error margin of ± 0.2 cm. The root mean square error of the thumb displacement data was also calculated and shown in Table 1.

**Table 1:** Root mean square error of the thumb data and net tilt angle during high force level and low force level grasp conditions. The table shows the average net tilt angle of the handle and root mean square error on the thumb normal force and displacement data during both grasp conditions. The mean and standard deviation (SD) of the data are presented.

Condition	RMSE of the thumb normal force data (N) (mean ± SD)	RMSE of the thumb displacement data (cm) (mean ± SD)	Net tilt angle (°) (mean ± SD)
High force level grasp	0.24 ± 0.05	0.09** ± **0.02	0.72** ± **0.24
Low force level grasp	0.36 ± 0.07	0.11** ± **0.01	0.78** ± **0.25

### Normal forces of the individual fingers during high force level and low force level grasp

**Figure 3:**
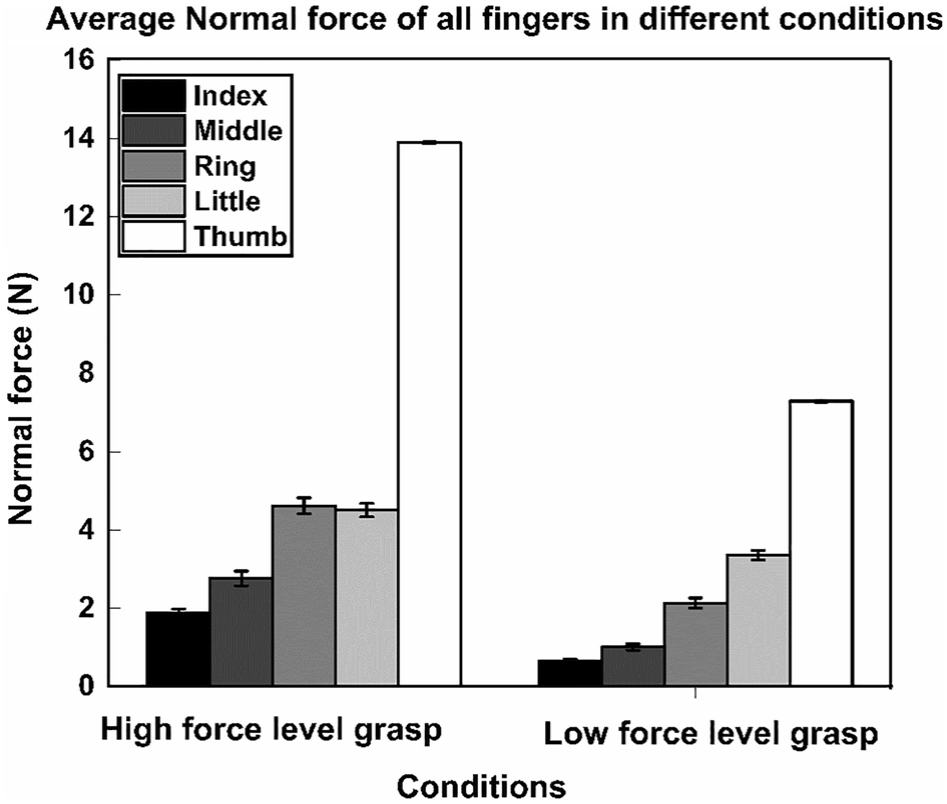
Average Normal forces of the individual fingers and thumb in different conditions. Little finger normal force (represented in light shaded gray) of high force level grasp condition was statistically equivalent to the ring finger normal force (represented in medium shaded gray) of the same condition. In contrast, during low force level grasp condition, little finger normal force (represented in light shaded gray) was statistically greater (p < 0.001) than the ring finger normal force (represented in medium shaded gray). During high force level grasp condition, the average thumb normal force (mean = 13.89 N, SD = 0.07) produced by the participants was statistically greater (p < 0.001) than the average thumb normal force (mean = 7.28 N, SD = 0.09) produced during low force level grasp condition.

During high force level grasp condition, the normal forces of the ring (mean = 4.61 N, SD = 0.70) and little (mean = 4.49 N, SD = 0.57) fingers were found to be statistically comparable (t(11) = − 3.207, p = 0.00418). This was confirmed by employing the TOST procedure with equivalence bounds of ∆_L_ (lower) = − 1.04 and ∆_U_ (Upper) = 1.04 for a desired statistical power of 95%. However, during low force level grasp condition, the normal force produced by the little finger (mean = 3.36 N, SD = 0.41) was statistically (p < 0.001) greater than the normal force produced by the ring finger (mean = 2.13 N, SD = 0.43) and thus supporting the mechanical advantage hypothesis (refer Fig. 3).

We performed a two-way repeated-measures ANOVA on the average normal force with the factors *condition* and *fingers* that showed the main effect of condition (F_(1,11)_ = 15,106.26; p < 0.001, η^2^_p_ = 0.99) corresponding to a statistically greater normal force for high force level grasp compared to low force level grasp. Similarly, the main effect of the factor *fingers* (F_(2.85, 31.35)_ = 81.264; p < 0.001, η^2^_p_ = 0.88) exhibited a statistically (p < 0.001) greater normal force by the ulnar fingers compared to the radial fingers.

The interaction *condition* × *fingers* (F_(3.12, 34.32)_ = 15.23; p < 0.001, η^2^_p_ = 0.58) showed a statistical effect reflecting the fact that the ulnar finger normal forces (Ring: mean = 4.61 N, SD = 0.70; Little: mean = 4.49 N, SD = 0.57) during high force level grasp was statistically greater (p < 0.001) than during low force level grasp (Ring: mean = 2.13 N, SD = 0.43; Little: mean = 3.36 N, SD = 0.41).

The post hoc pairwise Tukey test confirmed that the ring (mean = 4.61 N, SD = 0.70) and little finger (mean = 4.49 N, SD = 0.57) normal force of high force level grasp condition was found to be statistically greater (p < 0.001) than the radial finger (index and middle) normal forces of both high force level (Index: mean = 1.88 N, SD = 0.33; Middle: mean = 2.76 N, SD = 0.64) and low force level grasp (Index: mean = 0.65 N, SD = 0.16; Middle: mean = 1.01 N, SD = 0.28) conditions.

The task of maintaining the static equilibrium of the handle by producing a minimal normal force by the thumb along with the restriction to align the horizontal lines on the handle makes the task quite challenging.

During low force level grasp, the ulnar fingers normal forces (Ring: mean = 2.13 N, SD = 0.43; Little: mean = 3.36 N, SD = 0.41) was statistically greater (p < 0.001) than radial finger (Index: mean = 0.65 N, SD = 0.16; Middle: mean = 1.01 N, SD = 0.28) normal forces.

## Discussion

The main objective of the current study was to check and confirm whether the support for the mechanical advantage principle depends on the difficulty associated with the task. The little finger produced a greater normal force than the ring finger when the thumb was restricted to produce a normal force of 7 N (closer to the weight of the handle 6.86 N). We believed that the reason could be due to the demand associated with the task of maintaining the handle in static equilibrium by producing lesser normal force by the thumb. The cause and effect behind the results will be discussed in the following paragraphs.

Some of the studies in the past supported the mechanical advantage principle in certain conditions. In a five-finger prehension study, when a load of greater mass (2 kg) was suspended closer (1.9 cm) to COM of the handle, ulnar fingers exerted apparently comparable normal force^7^. However, when the same external load was suspended at a farther distance (7.6 cm) from COM, causing a greater moment, the little finger produced greater normal force than the ring finger. Similarly, in another multi-finger prehension study, MAH was supported even when a load of lesser mass (less than 2 kg) was suspended at a greater distance (8.9 cm) from COM of the handle^14^. Thus, this does not mean that the support for MAH is always dependent on the moment arm or mass of the suspended load or magnitude of moment requirement.

Our previous study on the systematic increase in the mass of the handle with the load suspended exactly below COM of the handle could help to understand this situation better^12^. Although external loads of mass ranging from 0.150 kg to 0.450 kg were suspended exactly below COM of the handle, MA principle was supportive only when an external load of mass 0.450 kg was added. From the results, it may be posited that the support for mechanical advantage principle could be due to the difficulty associated either with the mass of the suspended load, moment arm or magnitude of moment requirement.

The hypothesis was also supported when the thumb platform was made to operate in the region beyond the range of motion of carpometacarpal (CMC) joint of thumb during the pattern tracing study^15^. The study was comprised of two conditions: tracing trapezoid pattern and inverted trapezoid pattern. Depending on the condition, either trapezoid or inverted trapezoid pattern was displayed on the computer monitor. The task was to hold the handle with the unsteady thumb platform at the HOME position for a few seconds and translate the platform vertically towards the index finger side (during trapezoid condition) or little finger side (during inverted trapezoid condition). CMC joint of the thumb possesses a limited range of motion in the downward direction. Therefore, tracing the BOTTOM static portion of the inverted trapezoid pattern was quite challenging than tracing the TOP static portion of the trapezoid pattern. Although a greater compensatory torque was required due to the shift in the position of the thumb platform from HOME, the difficulty associated with operating the thumb beyond the range of motion of its CMC joint could also be the reason for supporting MAH.

In the current study, the task was made difficult by imposing restriction to produce low thumb normal force. Two constraints exist in common for both the experimental conditions of the present study. One was the constraint imposed on the handle design. That is, there were two different interfaces on the thumb side of the handle: the thumb-platform interface and platform-railing interface. Since the slider platform was mounted on the vertical railing fitted over the handle frame, the friction at the platform-railing interface was very low (µ ~ 0.001–0.002). Therefore, the tangential force produced by the thumb to hold the platform was maintained at a constant low magnitude (around 1 N). Secondly, throughout the entire trial, the slider platform had to be held at the HOME position by aligning the horizontal lines on the platform and the handle frame. Although these two constraints were common for both the conditions, third constraint of producing very low thumb normal force of 7 N (closer to the weight of the handle) makes the experimental conditions distinguishable from each other.

Since the tangential force exerted by the thumb was 1 N (as it had to hold the slider platform of mass 0.100 kg) for both the experimental conditions, the remaining tangential forces was shared by the rest of the fingers of the same hand. Due to the imbalance in the tangential force distribution between the thumb and the other fingers, pronation torque occurs in the anti-clockwise direction. However, to maintain the handle in the static equilibrium, compensatory supination torque was required. The thumb tangential forces and ring and little finger normal forces involve in the supination torque production. Since the thumb tangential force was maintained constant and minimal, it becomes the duty of the ring and little fingers normal forces to increase to produce compensatory supination torque. Sufficient amount of normal forces by the ring and little fingers would be required to counter-balance the tilt in the anti-clockwise direction. However, by increasing ring and little finger normal forces, the horizontal equilibrium of the handle would be disturbed. Therefore, to retain the horizontal equilibrium, thumb normal force increases significantly. Thus, the participants prefer to increase the normal force of the thumb, as a convenient and safer option. By this way, the participants hold the slider steady at the HOME position (matching the horizontal lines) and maintain the horizontal equilibrium of the handle. Thereby avoiding the ‘fear’ of slip of the slider platform downwards. On contrary, by restricting the thumb to produce low normal force (7 N), makes the task of maintaining the handle in static equilibrium quite difficult.

For the current study, the magnitude of target normal force to be produced by the thumb during high force level grasp condition, was chosen from the results of our previous study on the systematic increase in the mass of the handle^12^. As per the previous study, when there was no restriction on the normal forces, the average thumb normal force exerted (or preferred) by the participants was approximately 14 N when the total mass of the handle was 0.700 kg (same as in the handle used for the current study). Since there was no ‘fear’ of slip and tilt due to the exertion of high thumb normal force of 14 N, the task was found to be not demanding. The results showed a statistically comparable normal forces by the ulnar fingers. Therefore, for the current study, we expected that the ulnar fingers would continue to produce a statistically comparable normal force during high force level grasping. In align to the expectation, the results of the current study showed statistically comparable normal forces by the ring and little fingers when the target thumb normal force was set to 14 N.

In the case of low force level grasp condition, the target normal force was set to 7 N. Since the total mass of the handle with the external load was 0.700 kg, the total tangential force shared by the fingers and thumb for holding the handle, was approximately 6.86 N. Therefore, for low force level grasp condition, the instruction was to exert a minimal (or low) thumb normal force of 7 N (closer to the weight of the handle). Due to the target of setting low thumb normal force, the ulnar finger normal forces decreased. The decrease in the ulnar finger normal forces would be accompanied by a drop in the supination torque, as ulnar finger normal forces are contributors to supination torque. In response to this, there would be a pronation torque in the anti-clockwise direction due to the virtual finger tangential force. However, to maintain the rotational equilibrium of the handle, a sufficient compensatory supination torque was required without a substantial increase in the ulnar finger normal forces. Perhaps, by increasing both ring and little finger normal forces together, virtual finger normal force might increase, which might indirectly disturb the normal force produced by the thumb, as the thumb was restricted to produce lesser normal force. At such a situation, the participants experience the challenge of balancing both horizontal and rotational equilibrium. Thus, the task of maintaining the handle in static equilibrium by holding the movable thumb platform steady at the HOME position with minimal thumb normal force (7 N) was found to be demanding.

Hence, during low force level grasp condition, the participants merely focus to balance both by producing a sufficient supination torque without showing a greater increase in the total normal force of the ulnar fingers. Employing the mechanical advantage principle would be the best solution from the mechanics perspective. It involved increasing the normal force of the little finger than the ring finger. Thus, sufficient supination torque would be produced while simultaneously producing minimal total normal force. The support for MAH holds true not only due to the exertion of very high thumb normal force^12^ (16.50 N) (due to the addition of external load of mass 0.450 kg) but also due to the exertion of very low thumb normal force (7 N). Thus, this confirms that the difficulty associated with the task of maintaining the handle in static equilibrium contributes in supporting to Mechanical advantage principle.

It is possible to untangle the intricate details behind the results of both the conditions from an anatomical or biomechanical standpoint. The tendons of the extrinsic muscle, flexor digitorum profundus (FDP), extend to the distal interphalangeal (DIP) joints of the index, middle, ring, and little fingers. FDP muscle is responsible for the flexion of DIP joints of the four fingers and thus accountable for the normal force production in those fingers. Whereas the intrinsic muscles of the hand such as lumbricals, hypothenar, thenar, dorsal and palmar interossei muscles are involved in the precise (or dexterous) manipulation of the object^16–18^.

In the case of high force level grasp condition, since the thumb exerted a relatively high normal force of 14 N, extrinsic muscles responsible for forceful grip production would attempt to increase the virtual finger normal force. In particular, the forces of ulnar fingers increase more than the radial fingers (index and middle) due to the task requirement of compensatory supination torque. In the case of low force level grasp condition, since maintaining the handle equilibrium was quite challenging, dexterous control of ulnar finger normal forces was required for the minimal total normal force production and sufficient compensatory torque production. Among the ulnar fingers, the little finger has an additional group of intrinsic muscles referred to as hypothenar muscles (flexor digiti minimi, abductor digit minimi, opponens digiti minimi) in addition to the lumbrical muscle.

Since the little finger has the added advantage of a separate group of intrinsic muscles for the dexterous manipulation compared to the ring finger, CNS might have attempted to use the little finger compared to the ring finger as it has both anatomical and mechanical advantages. Hence, the little finger might have produced a greater normal force than the ring finger, supporting the mechanical advantage hypothesis. The unique muscle architecture of the little finger may be why the system chooses to employ the mechanical advantage principle, particularly when the task becomes challenging, as in the current study.

## Concluding comments

Maintaining the static equilibrium of the handle by producing the thumb’s normal force closer to the mass of the handle, which already has restrictions imposed on the thumb’s tangential force and position, makes the task quite challenging. Since the little finger has both anatomical and mechanical advantages, CNS might have decided to use the little finger to complete the task successfully. Thus, the challenge associated with the task had induced CNS to use the little finger, supporting the mechanical advantage hypothesis, by producing greater normal force in the little finger than the ring finger.

## Data Availability

The datasets generated and analysed for the current study was already published as a data descriptor^19^. The datasets are available in the figshare repository, https://doi.org/10.6084/m9.figshare.19207875.v4.
